# Genetic Diversity in Casein Gene Cluster in a Dromedary Camel (*C. dromedarius*) Population from the United Arab Emirates

**DOI:** 10.3390/genes12091417

**Published:** 2021-09-15

**Authors:** Abdullah Al Mutery, Naushad Rais, Walaa KE Mohamed, Tlili Abdelaziz

**Affiliations:** 1 Department of Applied Biology, College of Science, University of Sharjah, Sharjah P.O. Box 27272, United Arab Emirates; atlili@sharjah.ac.ae; 2 Molecular Genetics and Stem Cell Research Laboratory, University of Sharjah, Sharjah P.O. Box 27272, United Arab Emirates; 3 Human Genetics and Stem Cell Research Group, Research Institute of Sciences and Engineering, University of Sharjah, Sharjah P.O. Box 27272, United Arab Emirates; 4 School of Life Sciences, Manipal Academy of Higher Education, Dubai P.O. Box 345050, United Arab Emirates; naushad@manipaldubai.com; 5 Department of Genetics and Microbiology, Universitat Autònoma de Barcelona, P.O. Box 08193 Barcelona, Spain; walaakamal87@outlook.com

**Keywords:** Casein, *CSN1S1*, *CSN1S2*, *CSN2* and *CSN3*, camel, dromedary, next generation DNA sequencing, casein genetic variability, the UAE

## Abstract

Genetic polymorphisms, causing variation in casein genes (*CSN1S1*, *CSN1S2*, *CSN2*, and *CSN3)*, have been extensively studied in goats and cows, but there are only few studies reported in camels. Therefore, we aimed to identify alleles with functional roles in the United Arab Emirates dromedary camel (*Camelus dromedarius)* population to complement previous studies conducted on the same species. Using targeted next-generation sequencing, we sequenced all genes in the casein gene cluster in 93 female camels to identify and characterize novel gene variants. Most variants were found in noncoding introns and upstream sequences, but a few variants showed the possibility of functional impact. *CSN2* was found to be most polymorphic, with total 91 different variants, followed by *CSN1S1, CSN3* and *CSN1S2. CSN1S1, CSN1S2* and *CSN2* each had at least two variants while *CSN3* had only one functional allele. In future research, the functional impact of these variants should be investigated further.

## 1. Introduction

Dromedary camels (*C. dromedarius*) and camel milk production are of major economical relevance in the United Arab Emirates (UAE). Indeed, more than 250,000 milking camels exist in the UAE [1], with an estimated annual production of 40,000 metric tons of milk. Thus, camels have more than just cultural importance, as camel milk and its products have become increasingly popular in local and global markets as remedies for various pathological conditions such as diabetes [2] and inflammation [3]. A female camel may lactate over a period of 12–18 months, during which time it may produce 3–10 kg milk per day [4]. Milk production depends on various factors such as lactation stage, feeding conditions, and, most importantly, the breed. In camel milk, protein content can vary from 2.4–5.3%, with fat levels of around 3.1% [5].

Casein is a calcium phosphate-binding phosphoprotein of milk that exists in several molecular forms and is the main protein present in the milk. Camel milk proteins are affected by genetic polymorphisms as much as they are by environmental conditions. The four major casein proteins in camel milk are αs1-casein, αs2-casein, β-casein, and κ-casein, which are encoded by *CSN1S1*, *CSN1S2*, *CSN2*, and *CSN3*, respectively. In goats and cattle, these genes are mapped on chromosome 6 and span about 250 kb [6]. These genes have been characterized at the genomic [7], transcriptomic [8], and proteomic [9] levels. Furthermore, genetic polymorphisms and variants in casein genes have been reported in various animal species [10], including cattle [11,12,13], goats [14], and sheep [15], with goats and cows having the largest genetic variability. The identified variants have been associated with differential gene expression and rates of protein synthesis [14,15]. Moreover, the results of recent studies suggest that casein gene variants may be associated with milk yield and composition [16]. In camels, genetic variants have previously been reported for *CSN1S1* [17,18,19], *CSN2* [20], and *CSN3* [21]. Pauciullo et al. [22] performed comparative genome analysis on the camelids casein cluster and analyzed casein variability in Sudanese and Nigerian dromedary populations using single nucleotide polymorphisms. However, such studies are not available for dromedary camel populations in the UAE, and they represent an important contribution since as it was shown that Arabian camel-types have distinct genetic features from the African camel-types (32530038). Hence, the present study was undertaken with the primary objective of identifying genetic polymorphisms in all four casein genes by targeted next-generation sequencing technology.

## 2. Materials and Methods

### 2.1. Animals and Sample Collection

Blood samples (5 mL) were collected from each of 100 female *C*. *dromedarius* from different private farms in Dubai (Al Marmoon area) Supplementary Table S1. Whole blood samples were obtained from the jugular vein using a sterile needle by restraining the camels in a laying position. Following collection, the samples were kept on ice and delivered to the Integrated Scientific Solutions Laboratory (Dubai, UAE) within 1 h. The study complied with the ARRIVE guidelines and U.K. Animals Act 1986 and associated guidelines, and it was performed according to the guidelines of the Sharjah University Ethics Committee. 

### 2.2. DNA Extraction and PCR Amplification

DNA was extracted from 93 samples because 7 samples were lost during the shipment to the laboratory. DNA extraction was performed using 100 μL of anticoagulated blood and a DNeasy Blood & Tissue Kit (Qiagen, Dreieich, Germany) according to the manufacturer’s protocol. DNA was quantified via a NanoDrop 2000/2000c Spectrophotometer (Thermo Fisher Scientific, Waltham, MA, USA). Primers were designed for the required genes using Primer 3 Software with the reference accession IDs “NW_11591251.1” and “NC_009849.1” (primer sequences are shown in Table 1). The targeted genes were amplified by PCR using standard protocols with Prime Star *GXL* DNA polymerase (Takara Bio Europe, Göteborg, Sweden). After an initial denaturation at 95 °C for 30 s, 30 cycles of denaturation at 95 °C for 30 s, annealing. The primer specific Tm for 20 s, and extension at 68 °C for 40 s were performed followed by a final 5-min extension stage. The same PCR protocol and conditions were used for all of the amplification reactions. The amplicons were pooled in equimolar concentrations and subjected to 1× purification using Ampure XP beads (Beckman Coulter, Brea, CA, USA) per the manufacturer’s instructions. Details about the analyzed genes are reported in Table 2.

### 2.3. Next-Generation Sequencing

#### 2.3.1. Library Preparation and Sequence Data Generation

Libraries were prepared using a standard NEBNext Ultra DNA Library Prep Kit for Illumina (New England Biolabs) according to the manufacturer’s instructions; this process efficiently produces an increased yield of high-quality libraries from a broader range of input amounts (500 pg to 1 µg). Sequencing data were generated using an Illumina Platform (MiSeq) for 100 paired ends with a sequencing depth of >500× coverage.

#### 2.3.2. Data Analysis

Read quality was checked from fastq files using fastqc software according to base quality score distribution, sequence quality score distribution, average base content per read, GC distribution in the reads, PCR amplification issues, over-represented sequences, and adapter trimming. Based on the quality report from fastq files, we trimmed sequence reads where necessary to retain only high-quality sequences for further analysis, i.e., low-quality sequence reads were excluded from the analysis. The reads were filtered based on the base quality of Q20 in the initial cleaning step. The paired-end reads were aligned to the reference *Camelus* sequences downloaded from NCBI (NW_011591251.1), for which the primers were designed, using BWA (Version-0.7.12) (arXiv:1303.3997v1) with its default parameters. The aligned reads were first sorted using the Picard tool “SortSam” command and then the read duplicates were removed using the Picard “Mark Duplicates” command (http://broadinstitute.github.io/picard/ access on 3 December 2020). We used samtools [23] to identify single nucleotide variants and short indels. After calling the variants, we further filtered them to retain good quality variants (according to cut of quality ≥ 200 (QUAL) and alignment read-depth of 30, variant score, and other parameters). Functional annotation was conducted using snpEff [24], using the NW_011591251.1 reference genome. All the sequences were submitted to the Sequencing Reads Archive database and were released with BioProject accession number PRJNA682302

## 3. Results and Discussion

The whole casein gene cluster comprising four genes, *CSN1S1*, *CSN1S2*, *CSN2*, and *CSN3*, was sequenced in the DNA samples of 93 camels by next-generation amplicon sequencing. The casein gene cluster is located on chromosome 2q2.1 and its organization has been described previously [22]. On average, approximately 30 MB of data was generated for each sample. The raw data quality summary (available on request) showed that >96.94% of the total data met the ≥30 phred score criterion. The average A/T content was higher than the G/C content (approximately 60% vs. 40%), which has also previously been reported in the camel κ-casein gene [21] and seems to be the case among other species [25]. Variation in mRNAs and proteins is known to be primarily affected by alternative splicing, duplication, and insertion/deletion events, in addition to nucleotide mutations [26]. All such variations were observed in the present study; however, most were found in intronic regions while very few variations were either in the coding or promotor regions of genes with possibility of having functional or regulatory functions (Table 3).

### 3.1. Analysis of CSN1S1 Genetic Variability

Because genetic data on camelids is lacking, little is known about the diversity of *CSN1S1*. Exon skipping and duplication events previously reported in this gene [18] led us to investigate the genetic variability. Kappeler et al. [17] reported on the occurrence of two variants (A & B) for *CSN1S1*. In our study, 85 different variants were identified (Table 3). Most of these variants were in noncoding introns that were unlikely to affect protein function or expression. Only two missense variants and one splice site variant were found that could be expected to have functional effects (Table 4). The variant missense c.135T>G leading to p.Asp45Glu had a minor allele frequency (MAF) of 0.102 whereas c.70C>T resulting in p.Pro24Ser had a MAF of 0.054 (Table 4). Casein gene polymorphisms were earlier identified in different regions of Sudan; three protein patterns denoted α-casein A, C, and D were identified with major allele A frequency ranging from 0.75 to 0.90 in different ecotypes [27]. Compared with α-casein A and D at the gene level, the α-casein C variant shows a single G > T nucleotide substitution in exon 5 that led to a nonsynonymous amino acid exchange (p.Glu30 > Asp30). Our finding of two novel missense alleles opens the possibility of other forms of α-casein that have not yet been reported.

### 3.2. Analysis of CSN1S2 Genetic Variability

CSN1S2 has previously been shown to have high levels of conservation for unknown reasons [8]. In our study, we did not find any missense variants located in CSN1S2. However, we identified two variants that had not previously been reported: c.-19A>C, a promotor region variant with an MAF of 0.296, and c.403-9_403-4delTTTTCT, a splice site variant with an MAF of 0.323 (Table 3). The splice site variant had a strong influence on protein function and stability. Analysis of the camel promoter showed a TATA box located, with reference to the first nucleotide of the first exon, at nucleotides −32/−25 that was perfectly conserved among the compared sequences [8]. As the variant c.-19A>C was in the proximity of this region, it may have had an impact on the expression of this gene.

### 3.3. Analysis of CSN2 Genetic Variability

We identified 91 polymorphisms in CSN2 (Table 2), most of which were in introns and downstream noncoding regions. Only one synonymous variant, c.21C>A, was found in an exonic region; it resulted in the p.Ala7Ala change (Table 2). Pauciullo et al. [20] also reported this variant in Sudanese female camels, which was the first example of genetic polymorphism in the CSN2 coding region for such species. These authors also reported four additional polymorphic sites, one of which resulted in an amino acid substitution at the ninth triplet of exon 2 within the signal peptide. The translation initiation codon (AUG) and the DNA sequence immediately upstream and downstream are known to influence the translation process [28]. In mammals, a consensus sequence (GCCRCCAUGG) was indicated by Kozak [27] to be the optimal context for the initiation of translation, and the nucleotides −6, −3, and +4 (taking the A nucleotide of the AUG codon as number +1) are reported to be the most conserved positions in natural mRNA 17 sequences [27]. With the exception of the AUG (nucleotides: +1, +2, and +3), dromedary CSN2 showed only two nucleotides homologous to the sequence reported by Kozak [29], i.e., positions −3 and −1) [20]. In the present study, we observed upstream and downstream variants that were distant from these consensus sequences; hence, they were unlikely to have any functional consequences.

Many genetic variants of β-casein have been identified at the DNA or protein levels in domestic animals. For instance, cattle [12] and goats [11] are the most polymorphic species among ruminants, whereas pigs [30,31] and horses [32] are less polymorphic, and polymorphisms have not been detected in other investigated species such as donkeys and rabbits. Although camels have not been investigated extensively to date, the limited number of polymorphisms found in coding regions and the number of mutations detected in the remainder of CSN2 suggests that the genetic variability of camel β-casein is closer to that of nonruminants. However, β-casein is the most abundant protein component in camel milk [33,34] and any allele detected at the CSN2 locus might affect the quality and technological properties of milk (e.g., milk ethanol stability). Therefore, a full investigation of β-casein should be performed in camels to determine nucleotide variability and its potential influence on the quality and properties of camel milk.

### 3.4. Analysis of CSN3 Genetic Variability

CSN3 had the lowest number of variants compared with the other sequenced genes considering the *CSN1S1*, and *CSN1S2* together: 66 intronic variants and 9 upstream sequence variants. Only one variant showed a probable functional impact, i.e., a promotor region variant, c.-65T>C. T, which is a minor allele with an MAF of 0.296 (Table 2 and Table 3). The other upstream sequence variants, eight SNPs and one deletion, was observed to be very distant from the promoter region between −2161 to −4873. Hence, it is very unlikely that these would have any effect on the expression of the CSN3 gene. As well as the conserved TATA box reported by Pauciullo et al. [21] (see Section 3.2), six putative transcription factor binding sites were also almost perfectly conserved: one CCAAT enhancer binding protein-α at position −1025/−1016, one mammary gland factor/STAT5 at nucleotides −1018/−1008, one cAMP response element binding protein at −900/−891, two octamer binding proteins at positions −307/−299 and −277/−268, respectively, and one TATA binding protein at nucleotides −236/−227. The variant observed in our study, c.-65T>C, does not seem to affect any putative regulatory region. However, its closeness to the transcription start site suggests that it could affect gene expression. Thus, further investigation is required to verify the influence of the c.-65T>C allelic variant in camel CSN3 gene regulation.

## 4. Conclusions

The present study established the genetic variability of casein gene cluster in Arabian camels from UAE and identified novel genetic variants with possible functional impact. Future studies will be focused on the investigations of the functional role of the casein variants detected. For example, the impact on gene and protein expression might be evaluated. Furthermore, as the variants influence the milk composition, an extensive study correlating the presence of these mutations and milk quality (e.g., nutritional qualities) could represent a relevant contribution to the field. Finally, considering the genetic diversity between camel types (32530038), the detected variants might be tested as potential tools to breed classification.

## Figures and Tables

**Table 1:** Primers sequences.

Gene	Sequence (5′→3′)	Tm (°C)	GC (%)	PCR Product Sizes (bp)
*CSN2_F1*	ACAGGCAGGGCCAGTACTTT	61.1	55	5962
*CSN2_R1*	GACTCAGCTGGGTGTTGATTT	59.2	47.6	
*CSN2_F2*	CTGTTTGCTGCTGTTCCTCA	60.2	50	5031
*CSN2_R2*	CTCCCATCTGCACTTCCATT	60.1	50	
*CSN3_F1*	CTGGCCAAGGACTTCATAGC	59.8	55	6456
*CSN3_R1*	GGTTGTTCCTGGTTTTGCAC	60.4	55	
*CSN3_F2*	GGTGGCTATAAGTTGGCACA	58.7	50.0	5849
*CSN3_R2*	TTCTGACCAGGCCTCACTTC	60.4	55	
*CSN1S1_F1*	CCACCAAATTTTATAGGACAGC	57.7	40.9	7409
*CSN1S1_R1*	CCTACGCCACAACACTTTCA	59.8	50	
*CSN1S1_F2*	ATGAACTCAAGGCACCAACC	60	50	6343
*CSN1S1_R2*	CAGATATGGGTGGGAGGACA	60.7	55	
*CSN1S1_F3*	GCGAATGCTCCATATGCTTC	60.7	50	7265
*CSN1S1_R3*	GCAGGAGACCCAATGCTAAA	60.2	50	
*CSN1S2_F1*	GCATTCCGGGTCACATAACT	59.8	50	6767
*CSN1S2_R1*	TGCCTATACTGTGTTCCAAGCA	60.7	45.5	
*CSN1S2_F2*	ATTGCCTTCAACGTGGTTTC	60	45	6799
*CSN1S2_R2*	GTGTCAGAGCACACCTGGAC	59.3	60	
*CSN1S2_F3*	GCCAAGTGGGTAAGCACAAT	60	50	5716
*CSN1S2_R3*	GTGAGGCTAACAGGAAATGGAG	60.1	50	

**Table 2:** Summary of organization of CSN cluster in *C. dromedarius* as adopted from Pauciullo et al. [22].

Gene	Position	Size (bp) (A)	Intergenic Distance (bp) (B)	Total Size (bp) (A+B)	Exons
*CSN1S1*	242,112 to 258,587	16,476		16,476	20
*CSN2*	265,187 to 273,094	7908	6600 (*CSN1S1→CSN2)*	14,508	9
*CSN1S2*	321,355 to 335,898	14,544	48,261 (*CSN2→CSN1S2)*	62,805	17
*CSN3*	421,597 to 430,955	9359	85,699 (*CSN1S2→CSN3)*	95,058	5
Total		48,287		188,847	51

**Table 3:** Summary of total number of variants identified in the casein gene cluster.

Gene	Variant Type	Impact	Total Variants
*CSN1S1*	Intron variant	Modifier	76
	Missense variant	Moderate	2
	Splice region variant & intron variant	Low	1
	Synonymous variant	Low	1
	Upstream gene variant	Modifier	5
*CSN1S2*	3 prime UTR variant ^1^	Modifier	1
	5 prime UTR variant ^2^	Modifier	1
	Intron variant	Modifier	34
	Splice acceptor variant & splice region variant & intron variant	High	1
	Splice region variant & intron variant	Low	2
	Upstream gene variant	Modifier	3
*CSN2*	Downstream gene variant	Modifier	45
	Intron variant	Modifier	37
	Splice region variant & intron variant	Low	1
	Synonymous variant	Low	1
	Upstream gene variant	Modifier	7
*CSN3*	5 prime UTR variant ^2^	Modifier	1
	Intron variant	Modifier	66
	Upstream gene variant	Modifier	9

^1^ 3′ UTR is the portion of an mRNA from the 3′ end of the mRNA to the position of the last codon used in translation. ^2^ 5′ UTR is the portion of an mRNA from the 5′ end to the position of the first codon used in translation.

**Table 4:** Characteristics of genetic variants with functional impact identified in the casein gene cluster and their genotype and allele frequency distribution.

Gene	Variant	Genomic Impact	Functional Impact	Wild Type Homozygous (%)	Heterozygous (%)	Mutant Homozygous (%)	Minor Allele Frequency (Allele)
*CSN1S1*	c.135T>G	Missense variant	p.Asp45Glu	4.3	16.1	79.6	0.102 (T)
	c.70C>T	Missense variant	p.Pro24Ser	89.2	10.8	0	0.054 (T)
*CSN1S2*	c.-19A>C	5 prime UTR variant	Modifier	50.5	39.8	9.7	0.296 (c)
	c.403-9_403-4delTTTTCT	Splice site variant	Low	67.7 (del/del)	0	32.3 (ins/Ins)	0.323 (Ins)
*CSN2*	c.51+8T>A	Splice site variant	Low	49.5	37.6	12.9	0.317 (A)
	c.21C>A	Synonymous variant	(p.Ala7Ala)	49.5	37.6	12.9	0.317 (A)
*CSN3*	c.-65T>C	5 prime UTR variant		14.0	31.2	54.8	0.296 (T)

## Data Availability

Data available in a publicly accessible repository at https://www.ncbi.nlm.nih.gov/biosample/SAMN16987361, accessed on 3 December 2020 reference number PRJNA682302.

## Supplementary Materials

The following are available online at https://www.mdpi.com/article/10.3390/genes12091417/s1, Table S1: The ID, age, and relatedness of the Camels samples, the Camels found at the Elesly and Marmom farm in Dubai, UAE.
