# Supplementary material for: Genetic Diversity in Casein Gene Cluster in a Dromedary Camel (C. dromedarius) Population from the United Arab Emirates
Source: Genes (Basel). 2021 Sep 15;12(9):1417. doi: 10.3390/genes12091417 (PMC8465939; doi:10.3390/genes12091417)
Supplement: Supplementary file 1 [file genes-12-01417-s001.zip › genes-1322879-supplementary.pdf]

Supplement

Supplementary Table S1: The ID, age, and relatedness of the Camels samples, the Camels found at the Elesly and Marmom farm in Dubai, UAE

Camel ID	Age /Year	Related	Farm
C22	9	No	Lessly Farm
C23	9	No	Lessly Farm
C24	9	No	Lessly Farm
C25	7	No	Lessly Farm
C26	8	No	Lessly Farm
C27	7	No	Lessly Farm
C28	8	No	Lessly Farm
C29	9	No	Lessly Farm
C30	8	No	Lessly Farm
C31	8	No	Lessly Farm
C32	8	No	Lessly Farm
C33	9	No	Lessly Farm
C34	7	No	Lessly Farm
C35	6	No	Lessly Farm
C36	7	No	Lessly Farm
C37	7	No	Lessly Farm
C38	9	No	Lessly Farm
C39	10	No	Lessly Farm
C40	7	No	Lessly Farm
C41	6	No	Lessly Farm
C42	8	No	Lessly Farm
C43	8	No	Lessly Farm
C44	9	No	Lessly Farm
C45	6	No	Lessly Farm
C46	7	No	Lessly Farm
C47	7	No	Lessly Farm
C48	8	No	Marmoor Farm
C49	8	No	Marmoor Farm
C50	9	No	Marmoor Farm
C51	9	No	Marmoor Farm
C52	9	No	Marmoor Farm

C53	6	No	Marmoor Farm
C54	6	No	Marmoor Farm
C55	7	No	Marmoor Farm
C56	8	No	Marmoor Farm
C57	9	No	Marmoor Farm
C58	8	No	Marmoor Farm
C59	8	No	Marmoor Farm
C60	8	No	Marmoor Farm
C61	8	No	Marmoor Farm
C62	7	No	Marmoor Farm
C63	7	No	Marmoor Farm
C64	8	No	Marmoor Farm
C65	7	No	Marmoor Farm
C66	7	No	Marmoor Farm
C67	8	No	Marmoor Farm
C68	9	No	Marmoor Farm
C69	9	No	Marmoor Farm
C70	9	No	Marmoor Farm
C71	9	No	Marmoor Farm
C72	8	No	Marmoor Farm
C73	7	No	Marmoor Farm
C74	7	No	Marmoor Farm
C75	7	No	Marmoor Farm
C76	9	No	Marmoor Farm
C77	9	No	Marmoor Farm
C78	9	No	Marmoor Farm
C79	8	No	Marmoor Farm
C80	8	No	Marmoor Farm
C81	10	No	Marmoor Farm
C82	10	No	Marmoor Farm
C83	9	No	Marmoor Farm
C84	9	No	Marmoor Farm
C85	9	No	Marmoor Farm
C86	7	No	Marmoor Farm
C87	8	No	Marmoor Farm
C88	7	No	Marmoor Farm
C89	7	No	Marmoor Farm
C90	7	No	Marmoor Farm
C91	8	No	Marmoor Farm
C92	9	No	Marmoor Farm
C93	8	No	Marmoor Farm
C94	9	No	Marmoor Farm
C95	9	No	Marmoor Farm

C96	6	No	Marmoor Farm
C97	8	No	Marmoor Farm
C98	7	No	Marmoor Farm
C99	8	No	Marmoor Farm
C100	8	No	Marmoor Farm
C101	8	No	Marmoor Farm
C102	8	No	Marmoor Farm
C103	8	No	Marmoor Farm
C104	9	No	Marmoor Farm
C105	7	No	Marmoor Farm
C106	7	No	Marmoor Farm
C107	10	No	Marmoor Farm
C108	9	No	Marmoor Farm
C109	9	No	Marmoor Farm
C110	9	No	Marmoor Farm
C111	9	No	Marmoor Farm
C112	9	No	Marmoor Farm
C113	9	No	Marmoor Farm
C114	7	No	Marmoor Farm
C115	6	No	Marmoor Farm
C116	7	No	Marmoor Farm
C117	7	No	Marmoor Farm
C118	9	No	Marmoor Farm
C119	9	No	Marmoor Farm
